# Latest Developments in the Cast Gold Process

**Published:** 1909-03-15

**Authors:** Albert L. Le Gro

**Affiliations:** Detroit


### LATEST DEVELOPMENTS IN THE CAST GOLD PROCESS.

BY ALBERT L. LE GRO, D.D.S., DETROIT.

Read before the Michigan First District Dental Society, January 14, 1909.

The fundamental principles underlying the process of casting gold will, in my mind, ever remain the same and it is not the latest developments in the process that interests the practitioner of Dentistry most, but the latest developments in technique and with the permission of your honorable committee I will treat the subject from that standpoint.

It is not my purpose to write of the advantages that accrue to the average dentist should he adopt the method of casting gold fillings in his practice, for I assume that from its very incipiency even the most skeptical were quick to see how it would revolutionize certain operations for good in the profession.

At this stage in the development of appliances by manufacturers it is a matter of little consequence which machine you select though in my mind there is a difference which I will endeavor to show you later.

I have always advocated strict adherance to one method until that particular method had been mastered or found by personal experience, to be inadequate. If you are using one machine and your neighbor is using another, it does not necessarily follow that because your neighbor is getting better results than you, that his machine is necessarily to be credited with the success.

In a majority of cases, it is a matter of your neighbor mastering his machine earlier and at the same time developing a technique that is more careful and complete than yours.

Analagous cases are presented in other lines of machinery, such as automobiles for instance. Two men may have identical cars, one runs his with a minimum amount of upkeep and trouble and the other is always in trouble and finds that his way of taking care of and running his car is causing the expenditure of a large amount of money as well as time. I merely mention this to show that the man who is having trouble with a certain casting machine is by no means in an anomalous or unique position. He certainly has seen or heard of beautiful castings made by the same make of machine that he possesses, so the natural supposition is that he has left some little detail out in the process of casting which brings about his failure and he, not the particular machine, is to be blamed.

Before going into the subject of technique I would like to give you the results of my inquiries as to what ingredients should enter the make-up of a perfect inlay wax. I have found that a combination of Gum Demar, Carnabauer, and Paraffin in the proper proportions will make an ideal inlay wax that can be carved like a fine grade of clay without distorting, attendent fracture or the use of warm instruments, a method to be deplored, and forever banished in trimming wax inlays.

All except the Paraffin are waxes of vegetable origin and can be readily volatilized. The use of Bees Wax should be avoided as its addition even in much smaller quantities make a much softer wax which is more difficult to carve.

Waxes that contain animal extracts such as Stearin, Spermaceta, etc., should be avoided as they separate from the compound in heat, especially in a flame, and they tend to make the wax greasy and flakey. Carnabauer is the hardest and highest fusing of the waxes and is added to give the wax edge strength and hardness, and to make the compound higher fusing. The gum demar is a hard translucent gum which gives hardness and toughness to the compound. Demar overcomes the tendency to flake and scale which is characteristic of the wax compounds containing Paraffin. The addition of Paraffin makes a tough plastic compound which is easier to congeal. A small quantity of ceracin is used to prevent the wax from being brittle and a black vegetable coloring matter is added if desired. A vegetable coloring matter is recommended because it leaves no residue when the wax is burned out. Green should be avoided as most of the greens which are usable are salts of copper and poisonous. There is a great difference in Carnabauer wax on the market. Most of this wax is imported and used for making floor waxes and while you will find some that is hard as stone, most of it is crumbly and cuts more like an inferior grade of clay The best grade must be used for inlay wax as the quality of the wax depends largely on the quality of the Carnarbauer used. I know of no other wax on the market that suits me so well as that manufactured by the Ransom & Randolph Co., this comes very near to being just what we want. The busy dentist has little time to experiment with matters so small even though he should be thoroughly acquainted with the ingredients and quality of same, that enters into the makeup of such a compound.

That I may more clearly explain some of my theories as to cause and effect in casting gold it is necessary that I treat of the crystalization of the different grades of gold and the metallurgy of that metal to some extent also.
If you will take four nuggets of gold of 18-20-22 and 24 karats respectively and bring them to a dancing molten state and then quickly remove the flame, you will notice that the mass of pure gold ,in crystalizing retains practically the same shape in a crystalized form as it did in a fused state. The 22K will flatten some; the 20K. a little more and the 18K. will be perceptibly flattened. The natural deduction is that the molecules of the gold in the 24K. have the greatest tendency to retain a fixed relative position and as you go down the scale it becomes very necessary to use more definite pressure on lower grades than on the pure in casting.

The pure gold, the melting point of which is 2012 degrees Farenheit and much higher than the lower grades, necessarily crystalizes much slower than the others. About 30 seconds is necessary for the complete crystalization of pure gold; and right here I would like to tell you why I think some machines are better than others, even though beautiful work is accomplished with any of them. When molten gold is forced into a cavity, whether it be by air, centrifugal force or steam pressure, each molecule of the gold used is supposed to have a definite relative position. During the first few seconds in the process of crystalization, pressure should be definite to accomplish this, not increased or decreased and some means should be furnished by the manufacturers machines that admit of an intelligent understanding by the dentist of what is going on in the way of pressure during the entire process of crystalization. If a machine is not constructed on those lines it should at least admit of the slight increase of pressure from time of application until complete crystalization has taken place. In experiments conducted by myself about a year ago I learned that a definite pressure of 4 pounds, if absolutely definite during the entire time of crystalizing was infinitely more accurate than those that ranged from 30 pounds down, with no fixed pressure at any time. My own supposition is that the molecules were not held in their correct relative position during the entire period of crystalization and though the inlays were just as perfect to the naked eye, they did not fit into the cavity with the nicety that the one of 4 pounds definite pressure did. Even greater discrepency is shown in the use of different grades of gold. The common alloys of gold are silver and copper, either one of which tends to make the gold more sluggish than the pure gold. The lines in the finished casting of an alloyed gold are not as sharp and it is therefore not deemed advisable to use anything but pure gold in cavities of extreme shape for it is a rare case that presents no defects when the lower grades of gold are used. If alloyed gold must be used, I would suggest that gold alloyed with Copper or Platinum or both be used. Copper does not materially lessen the malleability of gold while at the same time it hardens it. Silver is objectionable at all times as an alloy for gold that is used in casting. It has been recently recommended by some well known dentist on account of the color produced, but it is a mistake to use gold alloyed with this metal, for aside from making the gold very sluggish, it has little edge strength and will not make what I call a perfect inlay in cavities of extreme shape.

With a little experience you can learn to distinguish the alloy in any gold combination on sight. A yellow tint generally indicates equal parts of Silver and Copper. A red tint, an excess of Copper. A green tint, an excess of Silver. One of the most desirable alloys for cast inlay work is composed of Gold, 22 Dwts. Plat. 18 Grs. Copper, 7 Grs. It is a rather difficult alloy to produce but makes a sharper casting than any other I have been able to find as there is very little oxidation compared with 18-20-and 22K. golds that are alloyed with silver and copper. I do not recommend this alloy for its ductility or malleability for like most of the alloys it is sadly deficient but is much superior to Gold alloyed with Silver as regards color and will make a sharper casting.

In regard to the latest developments in technique. There are a multiplicity of methods all of which it is not my purpose to treat. I will confine my remarks to the little hints that my own personal observation and experience have taught me. As you all know, the possibilities of this work are only limited by the skill and ingenuity of the man. So many different possibilities suggest themselves to different men in this work that it would be presumptuous on my part to attempt to tell you of all the new things that everyone has worked out.

In the May and June, 1908, number of the Items of Interest are two very interesting papers by Dr. Price, of Cleveland, which are well worth the time of any dentist and while the deductions of Dr. Price are not verified by my own personal experience there is much of interest in these articles that show thought and study. These articles come the nearest to working out the problem and are the only papers on casting gold that I have read that exhibit an intelligent knowledge of the conditions as they are.

SECTIONAL INLAYS—Used only in cavities of extreme shape where the caries has attacked the tooth to such an extent that it becomes necessary to retain all the solid tooth structure that is left. Take a cavity in a large molar approximal involving the occlusal angle with the diameter bucco-lingually much larger at the cervical than at the occlusal portion. The cavity is so large bucco-lingually at the cervical portion that in order to prepare a solid or one piece inlay, it would be necessary to cut down a considerable portion of good solid tooth structure. To avoid this the sectional inlays or two piece inlays are employed. These inlays should be made hollow by carving the wax inlay or by the use of suction and heat. I have been using a suction apparatus that is now on the market and while it is a great improvement over the carving it is quite crude. This defect will be overcome I have no doubt as the evolution of casting gold progresses in dentistry. The orifice of the hollow cavities should be so carved that where the two sections are in place the cement will form one solid mass running from the hollow in one section to the hollow in another.

Some one has suggested the use of Ethel Chloride on cotton for chilling wax inlays. I have found it very satisfactory also in rubbing a smooth surface on the outside of wax inlays. When the wax inlay is removed from the mouth it should be washed in soda water to remove the mucous which sometimes produces a surface on the gold inlay that you cannot account for otherwise.

I shall describe the Dittmar method for making a cast gold shell crown for I think it the most perfect gold crown ever constructed. If you must put on gold crowns and can get a good fee for them, the Dittmar method will enable you to portray nature as no other method will and at the same time enable you to construct a crown that will give the minimum amount of irritation to the gums. After the root and remaining part of crown have been ground down properly ,take 34 gauge pure gold, make band to fit. This gold is so thin and soft that an absolute fit can be burnished slightly under the gum. Trim band proper height for top to be soldered, which completes an ordinary box cap. Have the top and band fit accurately so that the minimum amount of 22K. gold, not solder, will be used to solder the two together. I recommend 22K. gold for soldering for without it, the gold used in casting, will melt a lower grade of solder and percolate or rather be forced in fine films up inside of the band. When cap is completed place on root and with modeling compound in a double articulating impression tray, take the bite and impression at the same time. Articulate plaster for articulating side and inlay investment compound for crown side and paint the adjoining teeth with oil. Now heat up a quantity of inlay wax and use camels hair brush to build up wax on the cap to proper countour and articulation, smooth surface with chloroform saw off adjoining teeth and then the waxed crown. Twist and cast. The possibilities here are great and most beautiful work can be accomplished.

GOLD AND TIN INLAYS.—To use an inlay made of a layer of tin and over it a layer of gold at first thought seems a good procedure but the only argument that I can see logical in its favor is the economy of gold. A combination tin and gold inlay can have no other particular advantage over a gold inlay for the layer of cement must come between the inlay and tooth substance and all the arguments used in favor of tin at the cervical one-third or one-half of the cavity are lost. The ideal procedure is to insert the tin with pluggers, burnish the top and then make a cast inlay of gold to finish the operation. To make a combination tin and gold cast inlay is somewhat difficult at least it has been so in my own experience. After preparing the cavity a wax inlay is made of the cervical one-third or one-half of cavity. The top of this wax is made smooth and then oiled. On top of this another wax inlay is made to come flush with borders of remaining portion of cavity and to restore occlusal and approximal contour. This part of wax is then removed and cast, leaving the cervical portion of wax still in cavity. When the occlusal portion is cast the gold is heated slightly, placed into position in the cavity and then withdrawn, when the cervical portion or remaining wax will be seen to be attached to the gold already cast. Now comes the most difficult part of the operation. Gold has a great affinity for tin and the flask holding the investment must be at about an exact temperature to cast the tin. Tin melts at 442 degrees Farhenheit and is not sensibly volatile so if the investment is heated until the wax is entirely burned out of the investment and there is no formation of gases continuing and then allowed to cool until about 400 degrees Farenheit. Melting the tin now with blowpipe using no more than the required heat of about 450 degrees, a successful casting on the gold inlay can be made.

If these instructions are not followed out, the gold under a greater degree of heat will entirely absorb the tin and a brittle lead color mass will be the result. Hydrochloric acid must not be used under any circumstances to pickel the filling as tin dissolves in the acid with the evolution of Hydrogen and formation of Stanuous Chloride.

Combination Gold and Porcelain Inlays.— One of the most satisfactory operations in large restorations is the gold inlay with porcelain face baked in. The inlay is made in the usual way preferably with Platinum alloyed gold. The wax inlay is carved out or sucked out with heat and air on the wax corresponding with the portion to be restored in Porcelain, with undercuts. After the inlay is made, Jenkins porcelain is baked in and an aesthetic result is accomplished. Before baking in the enamel, place gold inlay in cavity, burnish borders and reduce line of gold on exposed border between margin and cavity for porcelain to a minimum so that the line of gold will be almost imperceptible when work is finished.

PORCELAIN CROWN WITH CAST CAP AND DOWEL FOR BADLY BROKEN DOWN ROOTS.—This is a branch of the work of Drs. F. Ward Howlett and C. J. Lyons, of Jackson, Mich. They have worked together practically in producing a technique that gives very fine results in these extreme cases of badly broken down roots. I first saw the work of both these gentlemen, introduced at Indianapolis, early last year and after mastering it to some extent have gotten very satisfactory results in my own practice. An article on the subject, with detailed drawings by Dr. Howlett, appeared in the January issue of the DENTAL SUMMARY. Every dentist should read it. I will give briefly the technique:

HOWLETT'S CROWN.—A bite is taken over the abutment to be crowned with Stents modeling compound, which is very hard when cold. The compound is then carved to fill the space and imitate the crown to be restored. A thin platinum matrix is then swaged to the occlusal surface of the tooth which has been built up and carved from the compound. This matrix is then removed from the carved tooth and filled with porcelain by a series of bakings which results in a cap of porcelain such as might be stamped from gold in the die plate for a gold crown or bridge dummy. A second matrix of platinum is made on the root stump or abutment and porcelain built onto it restoring a considerable part of the tooth. This is then placed on the stump in the mouth and the occlusal part of the crown first made is waxed on top of it and the patient forces it to proper occlusion while the intervening wax hold the two sections of porcelain together and in place. The crown is then removed from the mouth and a portion of the wax removed and replaced with porcelain. Then the rest of the wax is removed and the space filled with porcelain and the whole crown is fused together into one piece.

Dr. C. J. Lyons crown which differs somewhat from that of Dr. Howlett's though the fundamentals are practically the same, is a porcelain crown with cast cap and dowel for badly broken down roots. Use iridio platinum post gauge 20 as a matrix for carrying wax into the root, letting the wax extend over the gingival margins of the root. This should then be carved to form a plate for the crown; a square post should be formed in wax about 5 milimeters long and set in the middle of the plate for the reception of the crown. The wax model of the cap and dowel is then cast in pure gold with the iridio platinum wire in the center, after which it is polished and set in the root. A platinum matrix is next made of the plate and post by forming a tube for the post and soldering to a square piece of platinum, afterwards it is burnished to a perfect adaptation to the plate and post. A Davis Crown is used for the body of the porcelain crown by grinding out a section from mesial to distal large enough to accommodate the post. This is waxed in position over the matrix and all withdrawn and Consolidated body packed around the matrix and fused. After the crown is completed the matrix is removed from the crown and the crown is cemented to the cap and dowel.

BRIDGEWORK.—Those who have had experience in casting bridges in one piece will agree with me that it is entirely the wrong procedure and especially where inlay abutments are employed. There is no known investment compound that when set even under perfect conditions, will expand enough to compensate for the contraction of gold when crystalizing . It is therefore necessary to cast bridges in sections and solder together in order to get exact results.

The fallacy of casting directly onto porcelain I think is quite well recognized. Such methods have no other claim to general adoption than that they are an easy and rapid method or means of obtaining a result which cannot be wholly successful.

In conclusion I will say that it has not been the object of the writer to assume that there is any "only way" in the processes of casting gold and while the subject given me by your committee is not one that I could possibly stick to the text on, my initial object will be accomplished if this papr but serves as a stimulus to bring out many instructive facts that you all must possess on this subject.

DISCUSSION.

DR. M. R. MUIR: Cast gold inlay work is not yet two years old, and everybody has his own little ideas, and we should have an expression from everyone here tonight who has had any experience in this work. I hoped to have some specimens to show you tonight, but I have only one which is a practical case that I made this afternoon,—a bicuspid crown with a porcelain face in it. The facing was removed and gold cast with lead pencil points in the pin holes to keep them open, and the facing is then attached by cement.

The subject of Dr. LeGro's paper must necessarily bring out the results of his own experience, and as it is largely original it is of much more interest than if he had obtained his ideas from books and magazines to which we all have access.

The latest processes in cast gold work are being developed by dentists every day in their own offices as the possibilities of the work done, by casting gold is as the essayist says, limited only by the ability of the operator. There has been comparatively little on this work in the dental journals in the last eight or ten months, nothing important during that time in the ITEMS OF INTEREST or the COSMOS, with the exceptions of the results of the work done by Dr. Price, as given in his splendid articles appearing in the May, June and December, 1908, ITEMS.

The seed was sown by Dr. Taggart when he addressed the Odontological Society, of N. Y., just two years ago to-morrow, giving to the profession the results of his labors and since that time it has taken root and flourished in nearly every dentist's office in the country. Numerous kinds of machines have been designed with a view to economy and the use of the cast gold inlay has been unmercifully abused by some. Dr. Hasley, in the January 1909 ITEMS says that "some of the machines are so far removed in form and action from the usual workroom appliances that until seen in operation they might pass for caricatures." One's thumb guarded by a wet rag has been found sufficient to force molten gold into a mold so as to produce an acceptable inlay. Compressed air, steam, gun powder, inertia centrifugal force and atmospheric pressure brought into action by an exhaust pump, a pill box, a child's toy tin pail swung around ones head, a pair of tongs, a yankee clock, an old fashioned coffee mill, and a hundred and one such like devices have been suggested in the dental journals for inlay castings.". Just here let me quote an expression of Dr. Price:—"You are disappointed just in proportion to the height of your ideals". Therefore I would say govern yourselves accordingly in selecting a machine.

I agree with the essayist in advocating a strict adherance to one method or machine that you know others to be getting good results with, stick to it yourself until you get results. Exactly the same can be said about inlay wax. I can get the same results with the wax I use that you can get with the wax you use and you can get the same results with the wax you use that I can get with the wax I use, which goes to prove that the wax for you to use is the wax that you know how to use.

There are several substitutes for pure gold which might be used in casting for anything but inlay work. My experience has been that nothing takes the place of pure gold for a perfect inlay. The only argument to be put up in favor of the alloy or any other material that might be used is economy, but I would not consider that, as the time consumed is the same and results are not as good, when alloys are used.

Sectional inlays are often employed to good advantage and are sometimes necessary to avoid unnecessary cutting. If there is an undercut and the overhanging walls are good and strong fill it with cement before taking the impression. After the sidewalls and margins are prepared, if there still remains decay in the bottom that if removed would make an undercut, take the impression and remove the decay after. A great many are of the opinion that grooves placed so and so in the cavity and in the inlay for the cement to lodge in help materially to hold the inlay in its place but I believe that the less cement you have between the inlay and the tooth the stronger and more permanent it will be. I had that ground into me by Dr. Reeves and it is still there.

On account of the perfect fit obtained with the cast gold inlay by burnishing, in certain cases with plenty of mechanical retention one can afford to sacrifice some of the strength given by the cement and I frequently hollow out large inlays with the Roach suction wax carver to save using so much gold or to make a layer of cement over the pulp to protect it from thermal changes. Your inlay will fit better and give better satisfaction if you will clean it with hydrofluoric acid after casting. A porcelain face may be baked into a gold inlay, or the cavity can be prepared in the inlay before setting and after it is set proceed in the usual way to make the porcelain inlay.

I do not know of a better way for chilling and removing wax inlays from the cavity than the following:—In the left hand hold a small abscess syringe with platinum point filled with cold water and with the right hand heat the end of a piece of copper wire about two inches long over a flame and insert it into the wax inlay—immediately then put a stream of cold water on the wire and the inlay—this holds the wire in its place. The water all going directly to the place where needed and the point of the syringe being small, the amount of water used is small. While cooling the inlay with the left hand pick up the pliers with the right, grasp the wire close to the inlay and remove. At this point clean it off with a cold air blast from your air syringe using from 10 to twenty pounds pressure. To remove the inlay from the wire, heat the wire in the center over a small flame and the inlay will fall off. A good wax inlay can easily be spoiled by hooking an exploring point into it and pulling and partly shoving it out as is necessary especially if it fits tightly as it should. I do not think there is a machine made that will cast a plate or bridge without getting some shrinkage, enough so that it will not fit; I have never seen any, and have tried it,—unless it can be done with Dr. Price's artificial stone. He claims great things for that, and I hope that he will be at our February clinic, and I will let him speak for himself at that time. That artificial stone, if it is not perfected now, I think will be some day, and will be the only method of casting large inlays, plates or bridges. A bridge can be cast separately, the dummies, and then the parts soldered together, but I do not think that there is a machine made that will cast a large bridge or plate that will fit.

Dr. J. M. Thompson. In regard to casting direct to the facings, some of us have done that, but we have never failed to find little cracks in the porcelain, which would contra-indicate that process. Dr. Howlett and Dr. Lyons' methods are very fine, but they contain a great many complicated points in technique. I have made one or two castings that impressed me with a simple way to get around all trouble with the porcelain, such as making it after the crown is finished from a matrix. In one case that I have in mind now I ground the molar to fit the teeth, articulated it as I wanted it, and in doing this I destroyed the original shape of the porcelain considerably. After forming it to just the size and shape I wanted it, I placed it in the furnace and restored the gloss; Then I took a piece of number 40 platinum foil and burnished it over the root surface which was to come in contact with the crown. I then forced an iridio-platinum post up into the canal through the 40 foil. Then I took the soft inlay wax and placed it in the root and warmed the wax, and also the porcelain, and had the patient bite right down upon the porcelain and force the wax into all the form prepared for it, the excess was then removed with an instrument,and the foil burnished up around the bottom of the porcelain. I then made the casting, and the porcelain went right back without any trouble whatever, and several that have seen it have pronounced it a pretty fair piece of work, and it seems to me that it simplifies the technique that Dr. Lyons and Dr. Howlett gave us in their papers, although they may have merit.

In this piece of work that Dr. Muir is showing tonight, I would like to ask him if he had to grind the surface at all where the facing fits.

Dr. Muir. I think you can cast just as well on porcelain as any other way, but I do not think it is advisable. I do not think it is necessary. In the first place, it changes the color so much. I have cast some on porcelain and have never cracked a tooth, but they have all been experiments, as I have never done a practical case in that way. I think they are just as strong, and I think the tooth has more and better protection, if the gold is cast on it than any other way. I do not see any necessity for checking a tooth while casting on it, but dont think it is desirable, because it is liable to change the color too much. I think it can be done just as well by cementing the facing after the casting is all complete.

I think I did have to grind the crown I am showing a little; I took just a little bit off the gold and ground the porcelain a little. There is some change due to a certain extent to the contraction of the gold. I do not think there is any machine made but what there is some contraction. In an inlay for a filling that can be taken care of by lapped margins and you can burnish it down so that you cannot detect the margin, but on a curved tooth as this is you have to grind a little off to allow the facing to go into that gold. It may have been due to the shrinkage of the gold, and it may have been due to the distortion. I did it hurriedly because I wanted to get it ready to bring over tonight. I do not think that is any argument against the use of the method, because that tiny bit of grinding can be done, by the articulating treatment.

Dr. M. L. Ward. Two points that the essayist brought out I would like to emphasize. The first one was the kind of gold. I was of the impression some time ago that only pure gold could be fused successfully. I am not certain now that it is not the only one which we can depend upon, but I cast day before yesterday a practical case out of 18K. solder, and have done it repeatedly, by cooling down the mould. It appears to me that the great trouble is not so much with the gold as with the condition of the gold and the temperature of the mould. Everybody has an idea that they must cast these inlays in a hot mould. In foundry work it is just the reverse, and, so far as I can see, it should be so here. The very best moulds are those which have been heated and then allowed to sit and cool down. 18-20-22K. alloys will cast pretty well if you allow the cast to cool. If you heat the gold with an ordinary blow pipe it does not cool down so rapidly, and the solidifying of the gold seems to be the cause of changing form. The casts that we now get the very best results from have been those that have been done in a mould which has been heated about fifteen minutes in an open flame and set aside to cool. If the gold was 22K. we cool more, and if 24K. we cool still more, so that when the gold strikes the mould it cools at once. A foundry man with whom I talked on the matter advocated taking a larger piece of gold than was actually required . The mould should be fed by a good bulk of gold, that is large enough to keep it molten.

I want to suggest that the size of the spruce wire is important, the one which I have adhered to closely is about

80..1,000, which is a little larger than a No. 14 wire. Another thing I have been doing is to measure the strength of investments in connection with the casting inlays, and the temperature at which they are weakest, and I find that those which are weakest are those which have been heated beyond ten or fifteen minutes. Almost all of them, one fundamentally a stronger one, will stand 200 degrees of heat fifteen minutes, and every minute after that the heat is applied the strength runs down, so that I can recommend most heartily the heating of them not longer than fifteen minutes, and then allowing them to stand fifteen minutes.

All alloys of gold are thicker after they have been heated once or twice. They take up or absorb gases and the molecules come closer together and stiffen the alloy. Pure gold will become thick after being heated three or four times. A good thing to correct that is to take pulverized potassium nitrate and borax in equal parts; direct the flame onto the gold and get it melted and put a little pinch of this mixture on and apply the flame again; do that probably eight or ten times. Keep the pipe going and dropping the potassium nitrate and borax on top. The oxygen, firing away like gun-powder, will loosen up the gold and let out the gases. If it is oxidized or carbonized it is done so by the potassium nitrate. This is a standard dry process for purifying gold. Everybody ought to have this potassium nitrate and borax, and after he has cast a few times the gold should be thus treated and it will become much finer and produce better castings. The investment material has given us a good deal of trouble and we shall probably have more of it for some time, but I think you have not treated it the way you should. Most investments have shrunken a little, some of them have been very weak, but you have not applied the fire to them properly.

DR. S. BECKER. I would like to hear from some one his experience with Acolite which I have used considerably for attaching porcelain crowns to badly decayed roots. I expected to find something in the paper on this material.

I have used it considerably. In fact, I have not put on a Davis crown since June without either a gold or Acolite fitting. I have used the gold for large crowns once or twice but it takes so much gold as to be prohibitive. Acolite has a good sharp edge but has no strength. I do not like to cast gold on the porcelain, for the reason that we are liable to check the porcelain because of the difference in expansion of porcelain, platinum pins in the tooth and the gold used in casting. I do not think Acolite has been generally used for so long but what we want to know some more about it . Many people cannot affort to pay for the gold, especially in attaching Logan crowns, where you will often find there is a big space on the lingual side. I have filled these spaces by casting direct to the porcelain, and with the Logan crown I can do it very nicely with the aco-lite, but whether that is going to stand I am not prepared to answer.

Dr. A. W. Dumas. I wish to speak in regard to the advisability of casting gold on to porcelain. I do not think any casting machine should take the credit of a successful casting of that kind; the credit should be given to the operator, who first heats his case and porcelain to the same degree of heat as the molten gold, because otherwise you have about the most powerful elements in nature, heat and cold, in contact, and unless you have your porcelain heated to the degree of your gold you are sure to have trouble. I have been successful in many cases in casting upon bare porcelain, but I think it is folly to take such a chance. I think it is wiser to take a piece of pure gold and anneal it and burnish it over the tooth; in that way you save time and will not crack your porcelain. The cracking of the porcelain I think is due to the platinum pin in the tooth. I believe that as soon as the molten gold strikes these pins there is an expansion of the pins which causes a fracture in the tooth. You may take a Davis crown, and you can cast your metal, gold or Acolite upon it without removing, cast the whole at once, and the metal will find its place into the inside of that crown so that it will be impossible for you to take it out. From that I should infer that the cracking of the porcelain was due mostly to the expansion of the platinum pin baked into the tooth. I think it is economy of time and everything to burnish a small amount of gold against your porcelain.

Dr. Muir. The credit should not be given to any machine for casting on the porcelain; it can be done with any machine. I do not advise anybody to do it. I say it can be done, but it is not desirable. If you will heat the case hot enough to get the porcelain so hot that the gold will not check it, it can be done in any machine. The facing has got to be hot for the same reason that when you cast on platinum or cast on gold plate, they have got to be hot so that the new gold will fuse onto them, and it will fuse onto them. This cast that I passed around has a platinum band. You cannot expect the hot metal to unite with a cold metal; the inside of the mould has got to be hot.

Dr. C. H. Land. I want to help you out a little on the casting. It is utterly impossible to fuse any kind of metal to a piece of porcelain. It was never known to happen. You can flow it over a piece of porcelain. Whenever a piece of porcelain and platinum come together, the difference between the shrinkage and the expansion of the porcelain causes the porcelain to crack from the stress on the pin. If you look at a piece of window glass upon which you see various designs, these are made by putting glue over the surface of a smooth piece of glass and allowing that glue to dry and in doing so it rips out the design. You can never fuse onto a vitreous glass or porcelain a metal that it does not change the same; it simply rips off the design. It is far stronger to cement a crown onto a metal base than to fire it on. Every Logan crown is weak in that respect. Every bit of porcelain is fused in the mass; and from the piece of metal inclosed we will always have stress upon it, so that the tendency is to burst when it is re-heated. Cement your crowns instead of fusing them. If there is no strain upon a piece of porcelain it will stand indefinitely if you do not fire-crack it. If you fire a piece of porcelain on a metal base as in continuous gum work it will change, carrying the metal plate with it, and the fractures are not enough to talk about; or it will peal off and leave a nice shining surface, and the thicker the metal the more it will fracture. To prevent fracture by the heat when soldering facings, before the gold is cold take a piece of asbestos fiber and cover the piece so that it cools slowly and it will not fracture. I have worked this hundreds of times and never had a fracture when I took that precaution.

DR. LE GRO. While I am pleased with the nature of the discussion tonight, I am somewhat disappointed. I thought, while listening to these discussions, very much of a few years back how we were all using Oxapara and did not know what we were using; only knew the result. It is much so with casting gold. I do not think that the dentists have any intelligent understanding of what is going on when they are casting gold. They know that they are getting results but do not know what the cause of it is. They have not gone into the metallurgy of gold, or effect of pressure, and that is something that we have got to get an intelligent understanding of before we reduce this to an exact science or practice. As I stated, we have a great many articles published in the different dental journals, there is but one man who has written a scientific paper, on this subject, and that is Dr. Price, of Cleveland. Of course he is in a position to go into this scientific research more than the rest of us. I am rather disappointed, therefore, that it was not more discussed from the scientific aspect. However, I expected an "experience meeting," and such meetings always give us much to think about.

